# Preclinical studies on the toxicology, pharmacokinetics and safety of K1-70^TM^ a human monoclonal autoantibody to the TSH receptor with TSH antagonist activity

**DOI:** 10.1186/s13317-019-0121-9

**Published:** 2019-11-07

**Authors:** Jadwiga Furmaniak, Jane Sanders, Jill Clark, Jane Wilmot, Paul Sanders, Yang Li, Bernard Rees Smith

**Affiliations:** AV7 Limited, FIRS Laboratories, Parc Ty Glas, Llanishen, Cardiff, CF14 5DU UK

**Keywords:** TSH receptor, Graves’ disease, Graves’ ophthalmopathy, Human monoclonal antibodies, Autoimmunity, Therapeutic antibodies, Toxicology

## Abstract

**Background:**

The human monoclonal autoantibody K1-70™ binds to the TSH receptor (TSHR) with high affinity and blocks TSHR cyclic AMP stimulation by TSH and thyroid stimulating autoantibodies.

**Methods:**

The preclinical toxicology assessment following weekly intravenous (IV) or intramuscular (IM) administration of K1-70™ in rats and cynomolgus monkeys for 29 days was carried out. An assessment of delayed onset toxicity and/or reversibility of toxicity was made during a further 4 week treatment free period. The pharmacokinetic parameters of K1-70™ and the effects of different doses of K1-70™ on serum thyroid hormone levels in the study animals were determined in rats and primates after IV and IM administration.

**Results:**

Low serum levels of T3 and T4 associated with markedly elevated levels of TSH were observed in the study animals following IV and IM administration of K1-70™. The toxicological findings were attributed to the pharmacology of K1-70™ and were consistent with the hypothyroid state. The no observable adverse effect level (NOAEL) could not be established in the rat study while in the primate study it was 100 mg/kg/dose for both males and females.

**Conclusions:**

The toxicology, pharmacodynamic and pharmacokinetic data in this preclinical study were helpful in designing the first in human study with K1-70™ administered to subjects with Graves’ disease.

## Introduction

K1-70™ is a high affinity human monoclonal autoantibody to the TSH receptor (TSHR) with the ability to block TSHR stimulation by TSH or TSHR stimulating autoantibodies [1–3].

K1-70™ was isolated from the peripheral lymphocytes of a patient with a history of autoimmune thyroid disease and as such represents a “natural” inhibitor of TSHR stimulation [1–3]. The currently available options for the management of thyroid over activity in Graves’ disease include anti-thyroid drugs, surgery or radioiodine ablation that are effective in the majority but not all of the patients [4–6]. Furthermore, there are limited specific therapies currently available for the management of Graves’ ophthalmopathy [7–9]. Targeting of the TSHR with K1-70™ may provide new therapeutic strategies for the management of patients with Graves’ disease, patients with Graves’ ophthalmopathy, patients with thyroid cancer and other patients who would benefit from controlling TSHR activity [10–15].

Preliminary in vivo studies have demonstrated that administration of K1-70™ causes biochemical hypothyroidism in untreated rats and in rats made hyperthyroid with the thyroid stimulating human monoclonal autoantibody M22 [16–18]. The decreases in thyroid hormone levels post K1-70™ were associated with thyroid follicular cell atrophy and enlarged thyroid follicles filled with colloid, consistent with hypothyroidism [17, 18].

A fully human recombinant K1-70™ IgG drug was produced according to current good manufacturing practice (cGMP) and the toxicity of K1-70™ was evaluated following weekly intravenous (IV) infusion or intramuscular (IM) administration to the rat and the cynomologus monkey for 29 days. An assessment of delayed onset toxicity and/or reversibility of toxicity was made during a 4 week treatment-free period. Furthermore pharmacokinetic profiles of K1-70™ in the rats and primates and the effects of K1-70™ on serum thyroid hormone levels in the study animals were also analysed. The study aim was to provide important toxicity, safety and pharmacokinetics information to design the first in human clinical trial with K1-70™.

## Materials and methods

### Study drug

Drug substance of recombinant human K1-70™ IgG batch number 376270 was produced and tested according to cGMP following industry standard procedures. The drug was formulated in 25 mmol/L sodium citrate; 75 mmol/L sodium chloride; 50 mmol/L glycine; 0.02% (w/v) polysorbate 80; pH 6.0 at 10 mg/mL concentration.

### Rat study set up

The study was conducted in accordance with the requirements of the Animals (Scientific Procedures) Act 1986 and local ethical approval.

RccHan:WIST rats were between 7 and 8 weeks of age at the start of dosing. Males weighed between 207.0 and 265.9 g and females weighed between 158.2 and 210.6 g. K1-70™ was administered via intravenous infusion (IV) or intramuscular injection (IM). Different dose levels were selected in the rat study: high dose (IV), intermediate dose (IV), low dose (IV), and low dose (IM*)*. A high IV dose (150 mg/kg/dose) was selected to provide an approximately tenfold exposure multiple over the calculated maximum exposure to be achieved in humans. A low IV dose (15 mg/kg/dose) was expected to mimic the highest human clinical dose. An intermediate IV dose of 50 mg/kg/dose was also included. For IV infusion the drug was delivered via a motorised infusion pump, into the lateral caudal vein. The IV route of administration was chosen as a possible human therapeutic route. For the administration of K1-70™ by IM injection, rats received 1 mg injection of K1-70™ into each thigh i.e. total of 2 mg/dose. The intramuscular route was added to assess the local tolerability and systemic toxicity of the IM route as this was intended as a clinical dose route. The control group of rats received IV infusions of vehicle only.

There were three subgroups of rats at all dosing levels for different study objectives. Subgroup 1 included the animals for the toxicity study and treatment-free (recovery) assessment; subgroup 2 was for the toxicokinetic study and subgroup 3 was for thyroid hormone level assessment (hormone group) (Table 1).

**Table 1:** Groups of RccHan:WIST rats for IV and IM administration of different doses of K1-70™

Group	Dose route	Dose level (mg/kg/dose)	Number of animals			
			Subgroup 1			
			Toxicity		Treatment free	
			Male	Female	Male	Female
1	IV	0 (control)	10	10	5	5
2	IV	15 (low)	10	10	–	–
3	IV	50 (intermediate)	10	10	–	–
4	IV	150 (high)	10	10	5	5
5	IM	2 mg per dose (low)	10	10	5	5

			Subgroup 2			
			Toxicokinetics			
			Male	Female		
1	IV	0 (control)	3	3		
2	IV	15 (low)	9	9		
3	IV	50 (intermediate)	9	9		
4	IV	150 (high)	9	9		
5	IM	2 mg per dose (low)	9	9		

			Subgroup 3			
			Hormone			
			Male	Female		
1	IV	0 (control)	5	5		
2	IV	15 (low)	5	5		
3	IV	50 (intermediate)	5	5		
4	IV	150 (high)	5	5		
5	IM	2 mg per dose (low)	5	5		

*IV* intravenous infusion; *IM* intramuscular injection

Subgroup 1 rats, (toxicity study; 10 animals/sex) were given 0 (control), 15, 50, or 150 mg/kg/dose by IV infusion or 2 mg/dose by IM administration. The rats were dosed once weekly on day 1, 8, 15, 22 and 29 and necropsied on day 30. The treatment-free animals (5 males and 5 females) received the control, a high 150 mg/kg/dose (IV) and a low 2 mg/dose (IM) only at the same time points as toxicity study rats, then were not dosed beyond the end of day 29 and had a four week treatment free (recovery) period. On day 30 of the recovery period (day 59 of the study) the animals were necropsied for assessment of delayed onset toxicity and/or reversibility of toxicity (Table 1).

Rats for the toxicokinetic study in subgroup 2 included 3 animals/sex dosed at 0 mg/kg/dose IV as the control, 9 animals/sex at 15, 50 or 150 mg/kg/dose by IV infusion and 9 animals/sex at 2 mg/dose by IM administration. The rats were dosed once weekly on day 1, 8, 15 and 22 (Table 1).

Hormone group rats (subgroup 3; 5 animals/sex) for the measurements of thyroid hormones were given 0 (control), 15, 50, or 150 mg/kg/dose by IV infusion or 2 mg/dose by IM administration. The rats were dosed once weekly on day 1, and 8 (Table 1).

Blood samples for haematology, coagulation and clinical chemistry were drawn from all toxicity and treatment free (recovery) rats (subgroup 1) at necropsy. Urine samples were collected overnight from all toxicity rats in week 4. Blood samples for toxicokinetic analyses were drawn from subgroup 2 rats at pre-dose, 5 min, 8, 24, 72 and 168 h after dosing on days 1 and 22 and the animals were terminated upon completion of blood sampling (day 29). Thyroid hormone levels were measured in blood samples from the hormone cohort (subgroup 3) rats taken on days 7 and 14 and in terminal samples from toxicity animals (subgroup 1) on day 30. The concentration of K1-70™ in serum samples was measured using the PK ELISA from RSR Ltd (www.rsrltd.com).

All rats were observed for signs of ill health or overt toxicity. The injection sites were examined approximately 1 to 2 h after dosing for any signs of inflammatory skin reactions.

Ophthalmic examinations were performed on all rats pre-treatment and on control rats, rats receiving the high IV dose and rats receiving an IM dose in week 4. Individual body weights were recorded pre-dose, day 7, twice weekly from day 1 (before dose) and day of (prior to) necropsy. Furthermore the amount of food consumed was determined weekly and consumption was calculated as g/animal/day.

Necropsies were performed for subgroup 1 rats on day 30 of the dosing phase and in the case of the recovery rats on day 30 of the treatment free phase (day 59 of the study). The organs were dissected free from fat and other contiguous tissue then weighed before fixation. A full macroscopic examination was performed and fixed tissues were examined microscopically by a pathologist.

### Cynomolgus monkey study set up

The study was conducted in accordance with the requirements of the Animals (Scientific Procedures) Act 1986 and local ethical approval.

The cynomolgus monkeys (*Macaca fascicularis*) were between 28 and 52 months old at the start of dosing. The K1-70™ was administered by IV infusion once weekly with dose volumes based on individual body weight. The animals for the toxicity study received the high dose of 100 mg/kg, the intermediate dose of 30 mg/kg and the low dose of 10 mg/kg of K1-70™, respectively. The K1-70™ was also administered to a group of toxicity animals via an IM injection of 2.5 mg into each thigh (5 mg/dose). The control group received IV infusions of vehicle only. Each toxicity group consisted of 3 male and 3 female animals and dosing was once weekly on day 1, 8, 15, 22 and 29. Animals for treatment free (recovery) assessments received only 0 (control), the high dose of 100 mg/kg IV or 5 mg/dose IM at the same time points as in the toxicity study, were not dosed beyond the end of day 29 and had a 4 week treatment free period prior to necropsy. The treatment free groups included 2 males and 2 females each (Table 2).

**Table 2:** Groups of cynomolgus monkeys of the *Macaca fascicularis* strain for IV and IM administration of different doses of K1-70™

Group	Dose route	Dose level (mg/kg/dose)	Number of animals			
			Subgroup 1			
			Toxicity		Treatment free	
			Male	Female	Male	Female
1	IV	0 (control)	3	3	2	2
2	IV	10 (low)	3	3	–	–
3	IV	30 (intermediate)	3	3	–	–
4	IV	100 (high)	3	3	2	2
5	IM	5 mg per dose (low)	3	3	2	2

*IV* intravenous infusion; *IM* intramuscular injection

All animals were observed for any signs of ill health or overt toxicity and were given a detailed physical examination at weekly intervals. The injection sites were examined for signs of any inflammatory skin reactions approximately 1 to 2 h after each dose. Ophthalmic examinations were performed on all animals pre-treatment and in week 4. Individual body weights were recorded once weekly from allocation to start of treatment, twice weekly from day 1 (before dose) and day of (prior to) necropsy and a visual appraisement of food consumption was performed daily.

Electrocardiography and blood pressure measurements were performed pre-treatment, in weeks 1 and 4 on a non-dosing day and also during the treatment free phase. Urine samples were collected from all animals pre-treatment and in week 4. Blood samples for haematology, coagulation and clinical chemistry were collected pre-treatment and in week 4. Blood samples for thyroid hormone analysis were taken pre-treatment, days 7, 14 and 22 of the dosing phase and day 28 of the treatment free (recovery) phase. The concentration of K1-70™ in serum samples was measured using the PK ELISA from RSR Ltd. Blood samples for toxicokinetics were taken, pre-dose, at 5 min and at 8, 24, 72 and 168 h after dosing on day 1 and day 22. A toxicokinetic sample was also collected on day 28 of the treatment free phase from the animals assigned to the recovery group.

Necropsies were performed on day 30 of the dosing phase and day 29 of the treatment free phase for all animals. The organs were dissected free from fat and other contiguous tissue then weighed before fixation. A full macroscopic examination was performed. After fixing, the tissues were examined microscopically by a pathologist.

### Pharmacokinetic analysis

Pharmacokinetic analyses were carried out using WinNonlin Phoenix Version 6.2.1, Tripos L.P [Previously Certara]. Noncompartmental analysis was applied to the individual serum K1-70™ concentration data for males and females. AUC_(0–t)_ refers to the area under the serum concentration–time curve calculated from 0 to t, where t is the time of the last measurable concentration, calculated by non-compartmental analysis using the log/linear trapezoidal rule. C_max_ depicts the maximum serum concentration determined by visual inspection of the concentration–time profile. T_max_ is the time of maximum concentration determined by visual inspection of the concentration–time profile. t_1/2_ is the half-life determined by linear regression of at least three data points (not including C_max_) on the log (concentration) vs time plot, with a correlation co-efficient (R^2^) of 0.9 or greater. Concentration values below the lower limit of quantitation (< 0.150 μg/mL) were treated as zero for statistical and pharmacokinetic analysis.

## Results

### Rat toxicology

**Fig. 1:**
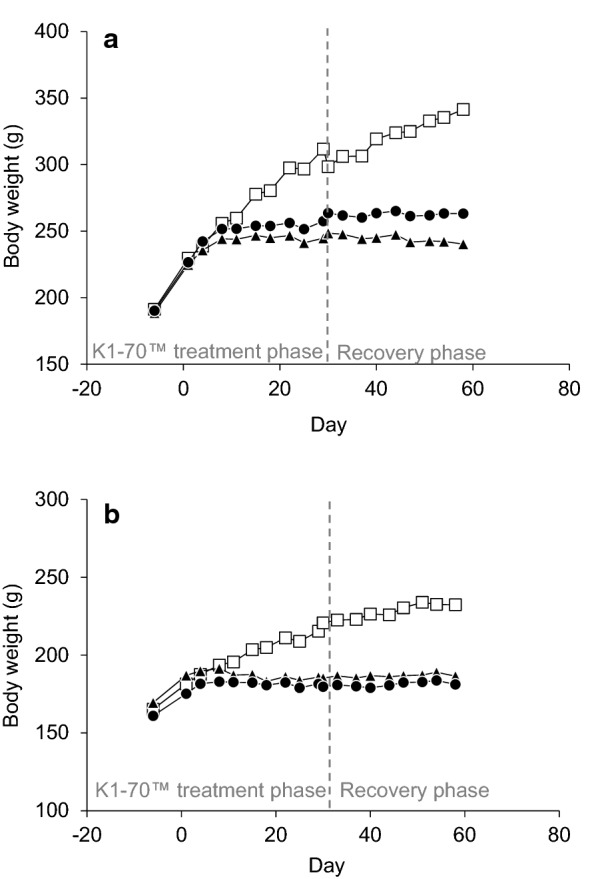
**a** Male rat body weights following K1-70™ treatment. Open squares = control; solid triangles = 150 mg/kg/dose IV; solid circles = 2 mg/dose IM. Reproduced with permission of the copyright holder, AV7 Ltd. **b** Female rat body weights following K1-70™ treatment. Open squares = control; solid triangles = 150 mg/kg/dose IV; solid circles = 2 mg/dose IM. Reproduced with permission of the copyright holder, AV7 Ltd

There were no unscheduled deaths; no clinical, post dose, or injection site observations; and no ophthalmic findings considered to be related to infusion or injection of K1-70™. Body weight gain and food consumption were low in both sexes in all K1-70™-treated groups with no recovery evident at the end of the 4-week recovery phase. The changes in body weight gain resulted in non-dose-dependent reduced mean body weights at the end of the dosing phase (approximately 17% to 22% lower in males and 13% to 16% lower in females) and at the end of the recovery phase (23% to 30% lower in males and 20% to 22% lower in females), relative to controls (Fig. 1).

After 5 doses of K1-70™, reticulocyte counts (absolute and percentage) were low and prothrombin times were long for all males treated with K1-70™; although no dose relationship was apparent. Platelet counts were high in males treated at 150 mg/kg/dose and 2 mg/dose with shorter activated partial thromboplastin times for females at the same dose levels. In all K1-70™ treated males cholesterol concentrations were high and alkaline phosphatase levels (ALP) were low and the effects were dose-dependent.

After 5 doses of K1-70™, total serum protein values were slightly higher in all males treated with K1-70™ compared to controls and were dose dependent; females given 150 mg/kg/dose IV also had raised total protein values. Creatinine and urea levels were high in all male and female groups. After 5 doses of K1-70™, there was no effect on urinary parameters.

### Rat thyroid hormone study

Levels of T4 and T3 on days 7, 14 and 30 were low compared to controls while levels of TSH were high compared to controls in all K1-70™-treated male and female groups with no recovery apparent at the end of the 4-week treatment-free phase. All male and female rats injected with K1-70™ were clinically hypothyroid by day 7 following one dose of 15 mg/kg, 50 mg/kg or 150 mg/kg IV or a 2 mg dose administered IM. No dose dependant effects were seen between the groups.

A summary of T4, T3 and TSH results on day 7 and day 30 of dosing are shown in Tables 3 and 4, respectively.

**Table 3:** Thyroid hormone levels in the study rats on day 7 post IV and IM administration of different doses of K1-70™

K1-70™ dose	Sex	TSH (μIU/mL)	T3 (nmol/L)	T4 (nmol/L)
		Mean ± SD	Mean ± SD	Mean ± SD
Control	M	0.60 ± 0.36	0.74 ± 0.12	78 ± 15.2
	F	0.49 ± 0.16	0.75 ± 0.20	56 ± 14.5
15 mg/kg/dose (IV)	M	15.62 ± 3.37	< 0.46^a^	< 26^a^
	F	18.30 ± 4.86	< 0.46^a^	< 26^a^
50 mg/kg/dose (IV)	M	16.82 ± 3.70	< 0.46^a^	< 26^a^
	F	18.24 ± 3.19	< 0.46^a^	< 26^a^
150 mg/kg/dose (IV)	M	15.32 ± 2.23	< 0.46^a^	< 26^a^
	F	16.20 ± 2.95	< 0.46^a^	< 26^a^
2 mg/dose (IM)	M	17.46 ± 4.93	< 0.46^a^	< 26^a^
	F	13.90 ± 3.08	< 0.46^a^	< 26^a^

Mean is of n = 5 animals per group per sex. One out of 10 rats injected IM with 2 mg doses of K1-70™ showed normal control levels of thyroid hormones 0.22 μIU/mL, 0.46 nmol/L and 61 nmol/L for TSH, T3 and T4 respectively and was not included in the range values. Total T3 levels in the serum were measured using the Siemens Centaur immunoassay system, and Total T4 and TSH levels were measured using Siemens Immulite system

*M* male, *F* female

^a^The concentration is below the detection limit

**Table 4:** Thyroid hormone levels in the study rats on day 30 post IV and IM administration of different doses of K1-70™

K1-70™ dose	Sex	TSH (μIU/mL)	T3 (nmol/L)	T4 (nmol/L)
		Mean ± SD	Mean ± SD	Mean ± SD
Control	M	0.39 ± 0.28	< 0.50^a^	55 ± 7.9
	F	0.18 ± 0.16	0.58 ± 0.09	< 33^a^
15 mg/kg/dose (IV)	M	40.36 ± 7.78	< 0.46^a^	< 26^a^
	F	35.21 ± 5.38	< 0.46^a^	< 26^a^
50 mg/kg/dose (IV)	M	39.06 ± 6.65	< 0.46^a^	< 26^a^
	F	39.62 ± 5.91	< 0.46^a^	< 26^a^
150 mg/kg/dose (IV)	M	36.41 ± 8.83	< 0.46^a^	< 26^a^
	F	33.23 ± 10.91	< 0.46^a^	< 26^a^
2 mg/dose (IM)	M	37.41 ± 14.42	< 0.46^a^	< 30^a^
	F	31.78 ± 13.32	< 0.46^a^	< 26^a^

Mean is of n = 10 animals per group per sex. One out of 10 rats injected IM with 2 mg doses of K1-70™ showed normal control levels of TSH 0.43 μIU/mL but T3 and T4 levels below the limit of detection and was not included in the TSH range value. Total T3 levels in the serum were measured using the Siemens Centaur immunoassay system, and Total T4 and TSH levels were measured using Siemens Immulite system

*M* male, *F* female

^a^The concentration is below the detection limit

### Rat histology

At terminal necropsy, group mean organ-to-body weight and organ-to-brain weight ratios in adrenal, kidney, heart and prostate weights were lower in treated rats when compared with concurrent controls, with no reversibility evident after the 4-week recovery phase. Macroscopic findings included pale/small thyroid; dark pituitary; thin uterus; and small prostate, seminal vesicles, testes, kidney, and liver; these findings showed no recovery.

**Fig. 2:**
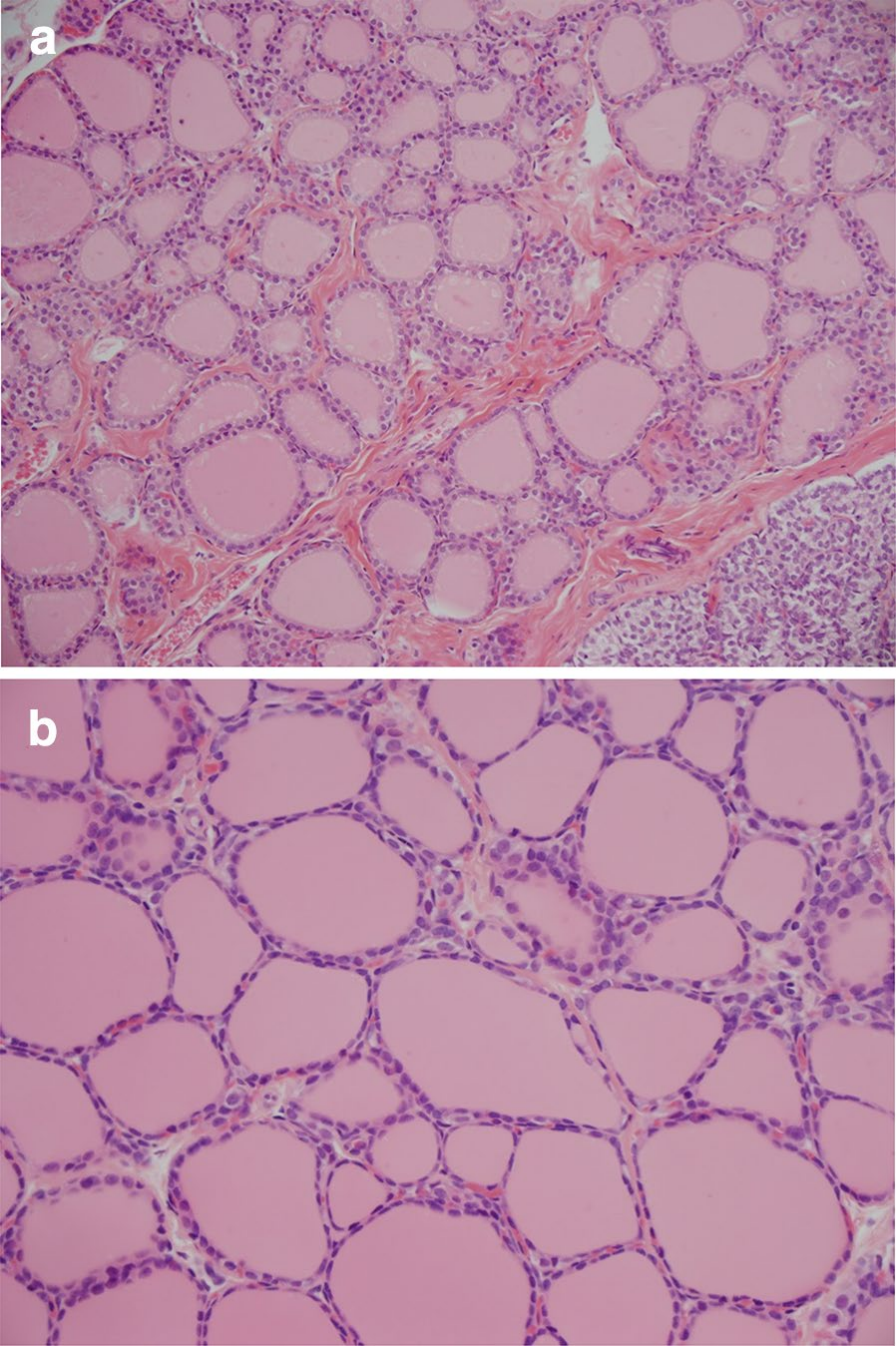
**a** Normal thyroid histology in a control rat (×20 magnification). Reproduced with permission of the copyright holder, AV7 Ltd. **b** Rat thyroid histology after K1-70™ treatment (×20 magnification). Follicular cell atrophy was recorded in most animals treated with all doses of K1-70™. Reproduced with permission of the copyright holder, AV7 Ltd

**Fig. 3:**
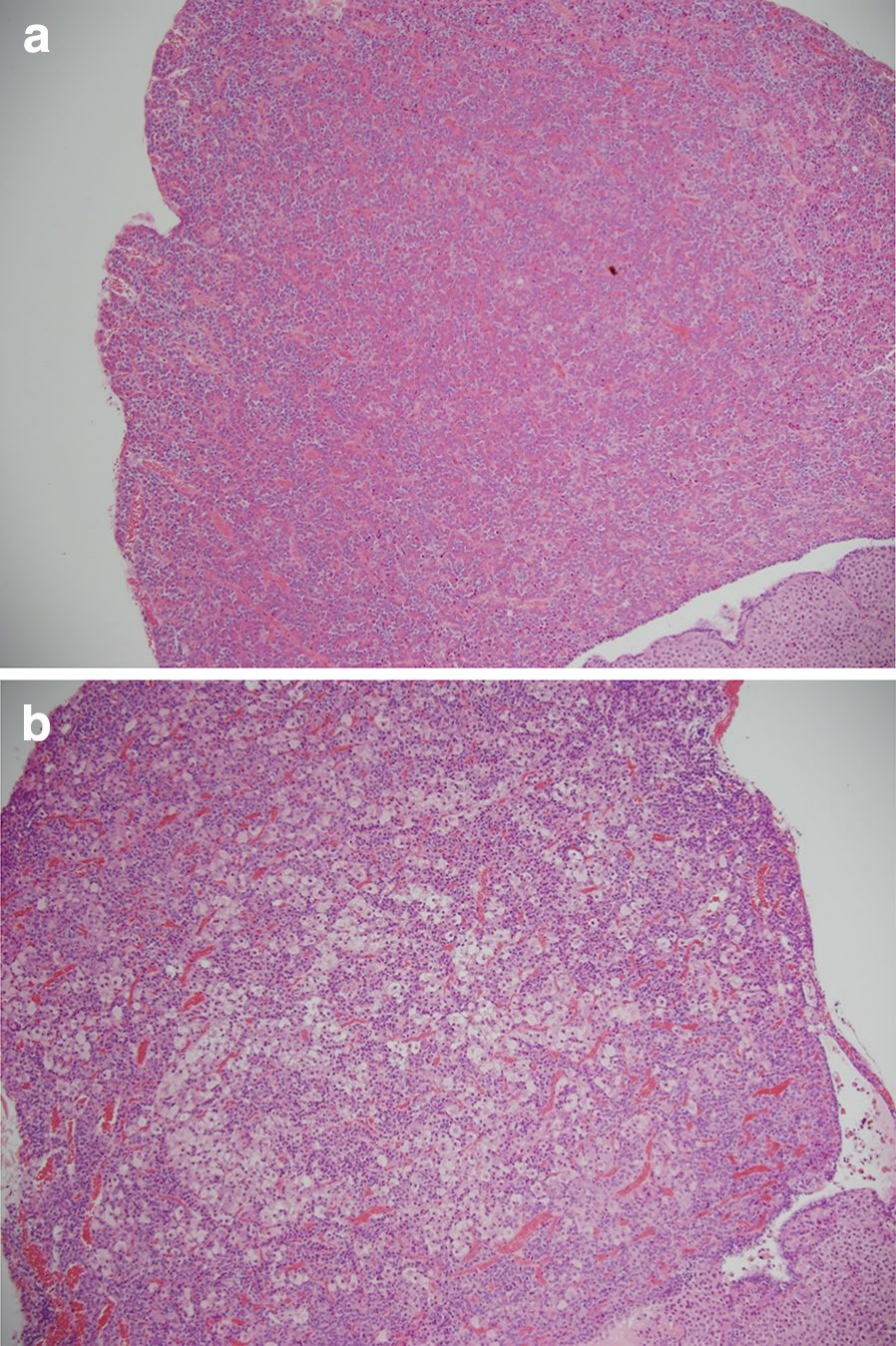
**a** Normal pituitary histology in a control rat (×10 magnification). Reproduced with permission of the copyright holder, AV7 Ltd. **b** Rat pituitary histology after K1-70™ treatment showing vacuolation in the pars distalis (×10 magnification). Pituitary vacuolation in the pars distalis was recorded in most animals treated with all doses of K1-70™. Reproduced with permission of the copyright holder, AV7 Ltd

Microscopic examination showed thyroid follicular cell atrophy in all animals treated with K1-70™ (except from one female in the IM group) characterised by follicles filled with pale-stained colloid and a reduction in height of the follicular epithelial cells without signs of inflammation (Fig. 2). After the treatment free period, follicular cell atrophy was recorded in 9/10 males and 9/10 females. There was no clear relationship to dose and no clear difference between animals dosed by IV infusion or IM injection. Furthermore, rat histology showed pituitary vacuolation in all animals treated with all doses of K1-70™ except for one female dosed with 15 mg/kg/dose IV and one female dosed with 2 mg/dose IM, and was generally more prominent in males. Vacuolation was characterised by enlarged cells in the pars distalis with increased cytoplasmic content, cytoplasmic vacuolation and eccentric or displaced nuclei (Fig. 3). This correlated with the dark pituitary noted on macroscopic examination. After the treatment free period, all treated males and 9/10 treated females were recorded with vacuolation in the pituitary. The histology findings in the thyroid and pituitary were consistent with a hypothyroid state and there was no evidence of reversal at the end of the recovery period.

Further histological findings consistent with a hypothyroid state were noted in the adrenal gland (cortical vacuolation), kidney (tubular basophilia, cortico-medullary mineralisation, and reduced hyaline droplets), testes (tubular atrophy), epididymis (oligospermia and cellular debris), seminal vesicle and prostate (contraction/atrophy), uterus (increase in number of females in dioestrus), femur and sternum (increased incidence of marrow fat), and brown adipose tissue surrounding the aorta and kidney (macrovacuolation). All findings showed little or no recovery at the end of the 4-week recovery phase.

### Rat toxicokinetic study

Following IV infusion, maximum serum concentrations of K1-70™ were reached at the end of infusion (5 min time point). Post C_max,_ serum concentrations declined with a long apparent mean half-life (range 104–263 h), which was independent of sex and dose. Exposure in male and female rats at the lowest tested IV dose (15 mg/kg) was an AUC_(0–t)_ of 82,400 and 83,100 µg h/mL and a C_max_ of 840 and 846 µg/mL, respectively, at the end of the study (week 4). Systemic exposure to K1-70™ (C_max_ and AUC_(0–t)_) increased in a manner broadly consistent with dose proportionality.

After IM administration, maximum concentrations of K1-70™ were reached between 24 and 72 h post dose, and there was evidence of a long-terminal half-life, but this could not be accurately characterized. Week 4 exposure after the 2 mg IM dose in male and female rats was 43,000 and 52,400 µg h/mL and a C_max_ of 274 and 333 µg/mL, respectively.

For both the IV and IM routes, based on C_max_ and AUC_(0–t)_ of K1-70™, accumulation was generally between two to threefold and consistent across dose groups, day, and sex and there was no evidence of a major sex difference. The toxicokinetic parameters are presented in Tables 5 and 6.

**Table 5:** Toxicokinetic parameters of K1-70™ in male and female rats following IV administration of different doses of K1-70™

Day	K1-70™ dose (mg/kg/dose)	Sex	C_max_ (µg/mL)	t_max_ (h)	AUC_(0–t)_ (µg h/mL)
1	15	M	273	0.6	20,800
		F	389	0.6	24,000
	50	M	1360	0.6	84,800
		F	1510	0.6	86,200
	150	M	4360	0.6	255,000
		F	4470	0.6	229,000
22	15	M	840	0.6	82,400
		F	846	0.6	83,100
	50	M	2630	0.6	255,000
		F	2610	0.6	267,000
	150	M	11,200	0.6	804,000
		F	6540	0.6	701,000

*AUC*_*(0–t)*_ area under the concentration–time curve over the interval 0 to t, where t is the time of the last measurable concentration, *C*_*max*_ maximum serum concentration, *F* female, *M* male, *t* 168 h, *t*_*max*_ time to maximum serum concentration

The level of K1-70™ in serum was measured using the PK ELISA from RSR Ltd

**Table 6:** Toxicokinetic parameters of K1-70™ in male and female rats following IM administration of K1-70™

Day	K1-70™ dose (mg/dose)	Sex	C_max_ (µg/mL)	t_max_ (h)	AUC_(0–t)_ (µg h/mL)
1	2	M	101	24	13,600
		F	130	24	18,300
22	2	M	274	24	43,000
		F	333	72	52,400

*AUC*_*(0–t)*_ area under the concentration–time curve over the interval 0 to t, where t is the time of the last measurable concentration, *C*_*max*_ maximum serum concentration, *F* female, *M* male, *t* 168 h, *t*_*max*_ time to maximum serum concentration

The level of K1-70™ in serum was measured using the PK ELISA from RSR Ltd

### Cynomolgus monkey toxicology

There were no unscheduled deaths during the study. There were no clinical, post dosing or injection site observations recorded considered to be related to treatment with K1-70™. There was no effect on body weight change, ophthalmoscopy findings, blood pressure, or organ weights. Throughout the 4-week study in monkeys, males given 100 mg/kg by IV infusion or a 5 mg IM dose had consistently higher bodyweights than other male groups including controls.

Following administration of K1-70™ at ≥ 30 mg/kg/dose IV to male monkeys there was a drug related decrease in heart rate, which correlated with the increase in RR interval at ECG in week 4. In the 30 and 100 mg/kg/dose groups, heart rate was 53 and 31 beats per minute (bpm) lower (respectively) than values in the vehicle-treated group and were decreased from pre-treatment values by 32 and 29 bpm (respectively) compared to no change in control animals following vehicle treatment. These changes were also evident in a single male following administration of 5 mg IM in week 4 which demonstrated a decrease in heart rate from pre-treatment (− 41 bpm) that was outside the range of values evident in the control group. Recovery to pre-treatment levels was demonstrated following the 4-week recovery phase.

There were no remarkable observations in haematology, clinical chemistry, coagulation and urinalysis in any of the animals treated with K1-70™.

### Cynomolgus monkey thyroid hormone study

High levels of TSH were detected over the duration of the study for males and females in all treated groups with males showing no recovery and females showing partial recovery at the end of the 4-week recovery phase (Tables 7 and 8). Serum T3 and T4 levels were low in all treated male groups throughout the study with no recovery evident. T3 levels were low throughout the study in all treated female groups with full recovery being shown at the end of the 4-week recovery phase for females treated at 100 mg/kg/dose IV and partial recovery for females at 5 mg/dose IM. T4 levels were low in the animals treated with K1-70™ and showed full recovery in females at the end of the observation period. A summary of these results is shown in Tables 7 and 8.

**Table 7:** Thyroid hormone levels in male and female monkeys post IV administration of different doses of K1-70™

Day	K1-70™ dose (mg/kg/dose)	Sex	TSH (µlU/mL)	T3 (nmol/L)	T4 (nmol/L)
			Mean ± SD	Mean ± SD	Mean ± SD
Predose	0	M	1.16 ± 0.46	2.35 ± 0.27	45.0 ± 4.6
		F	1.29 ± 0.54	2.43 ± 0.25	52.5 ± 15.8
	10	M	0.77 ± 0.57	2.04 ± 0.33	47.3 ± 7.6
		F	0.90 ± 0.38	2.91 ± 0.09	54.9 ± 2.6
	30	M	1.18 ± 0.94	2.75 ± 0.69	59.7 ± 2.7
		F	0.71 ± 0.11	2.65 ± 0.29	68.4 ± 9.4
	100	M	0.75 ± 0.39	2.31 ± 0.23	50.7 ± 6.6
		F	1.42 ± 0.61	2.93 ± 0.44	48.4 ± 10.8
Day 7	0	M	1.09 ± 0.42	1.75 ± 0.13	52.9 ± 6.9
		F	1.75 ± 0.55	2.15 ± 0.21	61.4 ± 11.4
	10	M	17.06 ± 4.23	0.83 ± 0.31	26.1 ± 8.4
		F	19.47 ± 11.69	0.62 ± 0.12	19.4 ± 1.3
	30	M	27.42 ± 6.78	0.67 ± 0.09	21.0 ± 3.9
		F	13.89 ± 2.96	1.01 ± 0.55	26.5 ± 8.8
	100	M	17.17 ± 7.00	0.87 ± 0.39	26.6 ± 8.9
		F	12.56 ± 7.65	1.18 ± 0.54	33.4 ± 15.3
Day 22	0	M	1.03 ± 0.35	1.96 ± 0.15	52.3 ± 7.2
		F	1.12 ± 0.27	2.19 ± 0.21	51.6 ± 5.8
	10	M	88.05 ± 24.46	1.03 ± 0.49	28.8 ± 12.3
		F	114.57 ± 2.47	0.79 ± 0.09	19.9 ± 2.0
	30	M	113.61 ± 4.13	0.63 ± 0.18	19.9 ± 2.1
		F	77.80 ± 66.16	1.43 ± 1.11	31.3 ± 17.8
	100	M	86.44 ± 17.03	0.93 ± 0.48	25.8 ± 11.6
		F	69.53 ± 48.45	1.64 ± 1.03	32.4 ± 15.0

For 10 mg/kg/dose and 30 mg/kg/dose, mean is of n = 3 animals per group per sex

For control (0 mg/kg/dose) and 100 mg/kg/dose, mean is of n = 5 animals per group per sex

Levels of thyroid hormones in serum, TSH, Total T3 and Total T4 were measured using Siemens Centaur immunoassay system

*M* male, *F* female, *T3* triiodothyronine, *T4* thyroxine, *TSH* thyroid stimulating hormone

**Table 8:** Thyroid hormone levels in male and female monkeys post IM administration of different doses of K1-70™

Day	K1-70™ dose (mg/dose)	Sex	TSH (µlU/mL)	T3 (nmol/L)	T4 (nmol/L)
			Mean ± SD	Mean ± SD	Mean ± SD
Predose	5	M	0.58 ± 0.20	2.45 ± 0.37	53.8 ± 12.2
		F	0.95 ± 0.56	2.75 ± 0.36	62.7 ± 11.8
Day 7	5	M	20.08 ± 9.30	0.96 ± 0.20	27.4 ± 8.6
		F	23.03 ± 10.44	0.84 ± 0.28	22.5 ± 3.3
Day 22	5	M	78.69 ± 35.66	1.18 ± 0.45	28.2 ± 13.2
		F	103.97 ± 26.90	1.21 ± 0.61	24.4 ± 4.9

Mean is of n = 5 animals per group per sex

Levels of thyroid hormones in serum, TSH, Total T3 and Total T4 were measured using Siemens Centaur immunoassay system

*M* male, *F* female, *T3* triiodothyronine, *T4* thyroxine, *TSH* thyroid stimulating hormone

### Cynomolgus monkey histology

Macroscopic examination at necropsy revealed small thymus in all treated groups, particularly in males given 30 or 100 mg/kg/dose IV. At the end of the 4-week recovery phase, the macroscopic findings in the thymus were comparable to the controls. Microscopically, in the thymus there was evidence of involution/atrophy in treated groups; particularly in animals dosed IV where there was a dose–response. This was characterised by the loss of lymphoid cells from both the cortex and medulla with reduction in overall size of thymus, and generally correlated with the small size noted macroscopically.

**Fig. 4:**
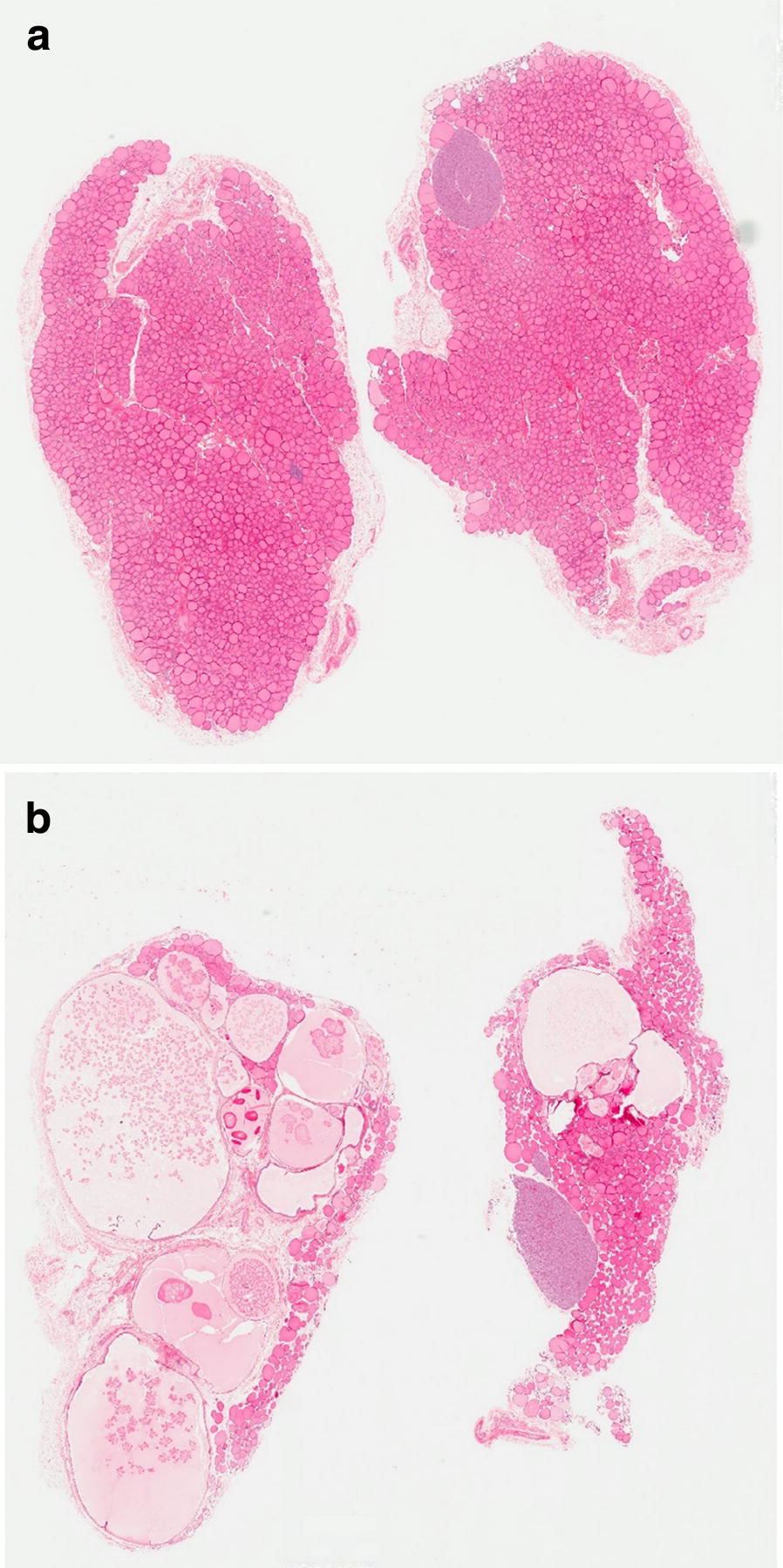
**a** Normal thyroid histology in a control cynomolgus monkey (×0.7 magnification). Reproduced with permission of the copyright holder, AV7 Ltd. **b** Cynomolgus monkey thyroid histology after K1-70™ treatment (×0.6 magnification) showing thyroid cystic follicles. There was increased severity of cystic follicles in the thyroids of male cynomolgus monkeys treated IV with K1-70™. Reproduced with permission of the copyright holder, AV7 Ltd

**Fig. 5:**
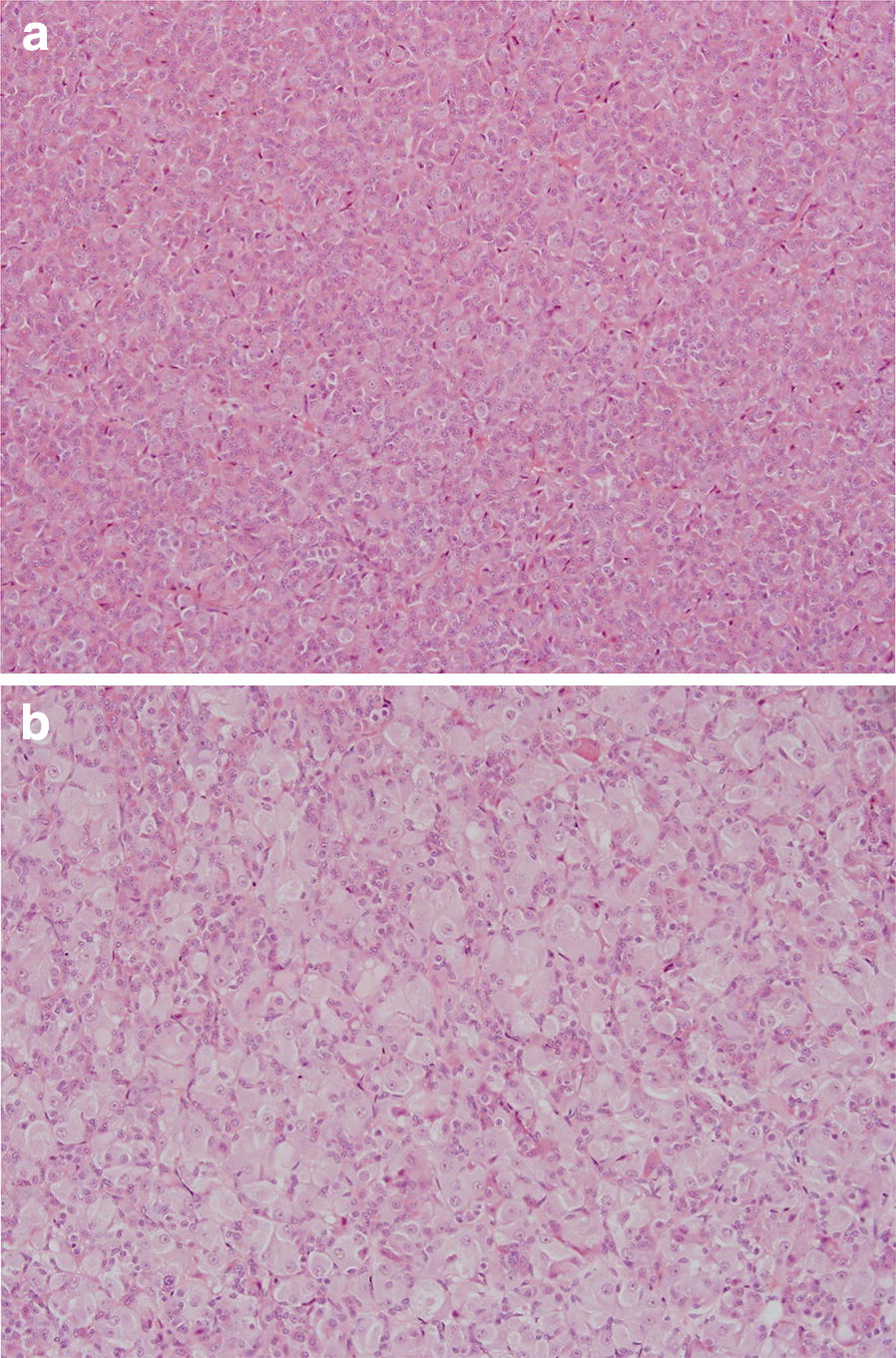
**a** Normal pituitary histology showing the pars distalis in a control cynomolgus monkey (×20 magnification). Reproduced with permission of the copyright holder, AV7 Ltd. **b** Cynomolgus monkey pituitary histology after K1-70™ treatment showing vacuolation in the pars distalis (×20 magnification). Pituitary vacuolation in the pars distalis was recorded in most animals treated with all doses of K1-70™. Reproduced with permission of the copyright holder, AV7 Ltd

On microscopic examination of the thyroid there was an increased incidence and/or severity of cystic follicles in males treated with K1-70™ via IV infusion without a clear dose–response effect. This was characterised by the presence of enlarged/distended follicles lined by normal epithelium (Fig. 4). At the end of the 4-week recovery phase cystic follicles were no longer present in thyroids of IV treated males. In the pituitary there was vacuolation recorded in most animals treated with all doses of K1-70™ without reversal at the end of the recovery (Fig. 5).

### Cynomolgus monkey toxicokinetic study

Following an IV infusion of K1-70™ to male and female monkeys at 10, 30 and 100 mg/kg, median serum concentrations were generally attained at the end of infusion on day 1 and day 22 although females at 10 and 30 mg/kg on day 22 showed median values at 8.5 h. Post C_max_, serum concentrations declined with a long apparent mean half-life of about 200 h, which was independent of dose, sex and day. The no-observed adverse effect level (NOAEL) was considered to be 100 mg/kg/dose IV for both males and females equivalent to an AUC_(0–t)_ of 595,000 and 592,000 µg h/mL and a C_max_ of 5610 and 5560 µg/mL, respectively, at the end of the study. Systemic exposure to K1-70™ (C_max_ and AUC_(0–t)_) in males and females increased in a manner broadly consistent with dose proportionality and there was no evidence of a sex difference (Table 9). Accumulation was in the region of 2.5-fold and consistent across dose groups, day and sex.

**Table 9:** Toxicokinetic parameters of K1-70™ in male and female monkeys following IV administration of different doses of K1-70™

Day	K1-70™ dose (mg/kg/dose)	Sex	C_max_ (µg/mL)	t_max_ (h)	AUC_(0–t)_ (µg h/mL)
1	10	M	275	0.6	27,000
		F	348	0.6	28,900
	30	M	1090	0.6	97,500
		F	1000	0.6	93,800
	100	M	2820	0.6	254,000
		F	3180	0.6	268,000
22	10	M	688	0.6	64,100
		F	597	8.5	66,900
	30	M	1910	0.6	195,000
		F	1710	8.5	173,000
	100	M	5610	0.6	595,000
		F	5560	0.6	592,000

The level of K1-70™ in serum was measured using the PK ELISA from RSR Ltd

*AUC*_*(0–t)*_ area under the concentration–time curve over the interval 0 to t, where t is the time of the last measurable concentration, *C*_*max*_ maximum serum concentration, *F* female, *M* male, *t* 168 h, *t*_*max*_ time to maximum serum concentration

After IM administration to male and female monkeys at a fixed dose of 5 mg, maximum serum concentrations were reached between 24 and 72 h post-dose. There was evidence of a long-terminal half-life, but this could not be accurately calculated. Week 4 exposure in males and females after the 5 mg IM dose was 6620 and 7490 µg h/mL, and C_max_ of 56.8 and 48.5 µg/mL, respectively. Similar to the IV route, based on C_max_ and AUC_(0–t)_ of K1-70™, accumulation was approximately 2.5-fold on day 22 and there was no evidence of a sex difference (Table 10).

**Table 10:** Toxicokinetic parameters of K1-70™ in male and female monkeys following IM administration of K1-70™

Day	K1-70™ dose (mg/dose)	Sex	C_max_ (µg/mL)	t_max_ (h)	AUC_(0–t)_ (µg h/mL)
1	5	M	13.3	72.0	2020
		F	16.9	72.0	2600
22	5	M	56.8	24.0	6620
		F	48.5	24.0	7490

The level of K1-70™ in serum was measured using the PK ELISA from RSR Ltd

*AUC*_*(0–t)*_ area under the concentration–time curve over the interval 0 to t, where t is the time of the last measurable concentration, *C*_*max*_ maximum serum concentration, *F* female, *M* male, *t* 168 h, *t*_*max*_ time to maximum serum concentration

## Discussion

In the rats, administration of K1-70™ resulted in low serum levels of T3 and T4 and high levels of TSH (Tables 3 and 4) which were the expected pharmacodynamics effects. Findings of thyroid follicular cell atrophy were considered directly related to the action of K1-70™ causing hypothyroidism via effective blocking of TSHR stimulation by TSH [19] and were consistent with the low thyroid hormone levels recorded in the study animals (Tables 3 and 4). The expected increased secretion of TSH by the thyrotropic cells in the pituitary in response to low thyroid hormone levels [19] was consistent with the markedly elevated TSH levels noted in rats in this study (Tables 3 and 4) and the finding of enlarged vacuolated cells in the pituitary.

Furthermore, the macroscopic and microscopic findings seen in other tissues were consistent with the hypothyroid state and the pharmacological action of K1-70™. Reduced weight of adrenals as observed in this study was consistent with the effects of hypothyroidism in rats and reported increase in pituitary content of adrenocorticotropic hormone in hypothyroid rats which could also contribute to increased pituitary vacuolation seen in the study animals [20]. The small kidney and reduced kidney weights noted in rats at the end of the dosing phase necropsy and the elevated serum creatinine levels were consistent with reported kidney derangements associated with hypothyroidism [21]. The reduction in hyaline droplets [22] in treated males was probably associated with altered renal metabolism or excretion, but due to the metabolic effects of thyroid hormone, an effect on protein metabolism cannot be excluded. In addition, as the high serum creatinine and urea levels were attributed to hypothyroidism [21], they were therefore, pharmacodynamic effects of K1-70™.

The reduced heart weight seen in the rats in this study was consistent with the effect of hypothyroidism and may occur in the absence of histopathological changes in the heart [23]. Furthermore, hypothyroidism is also associated with dysfunction of the gonads [20]. Although there was no clear effect on the testis or ovaries, minor tubular atrophy in the testis of 2 rats at the end of dosing phase necropsy and in 2 rats at the end of the recovery phase necropsy and in a clustering of females in metoestrus/dioestrus is suggestive of an effect via disruption of the pituitary–gonadal axis secondary to hypothyroidism. The same mechanism is likely to be responsible for prostate and seminal vesicle atrophy seen in the rats in this study [24].

Hypothyroidism causes changes in the handling of long chain fatty acids by white and brown adipocytes. In brown adipocytes fatty acid synthesis from glucose is substantially enhanced in hypothyroidism [25]. In this study, the large clear vacuoles seen in the cytoplasm of brown adipose tissue in the rats were consistent with lipid storage vacuoles, which in turn was consistent with enhanced fatty acid synthesis.

However TSHR is also expressed in multiple extra thyroidal cells including fat, kidney, bone and testis [26–30] therefore a direct relationship of the macroscopic and/or microscopic findings in different tissues to K1-70™ cannot be completely excluded.

Low body weight gain, body weight, and food consumption were considered to be related to treatment with K1-70™. Changes such as low reticulocyte counts, increased cholesterol and decreased ALP were considered to be related to the body weight and food consumption effect. Increases in total protein were considered to be reflective of dosing with a protein and, therefore, of no toxicological significance. The rats selected for the study were of young age to avoid the effects of the oestrus cycle on the study, and were still growing during treatment with K1-70™. Consequently the effect of hypothyroidism on growth and body and organ weight was marked.

Long prothrombin times were noted in male rats following administration of K1-70™. This was considered a secondary effect of hypothyroidism and has been described in humans [31, 32]. As this finding was associated with changes in platelet counts in males and activated partial thromboplastin time in females, the toxicological relevance of this finding cannot be discounted.

At the end of the recovery phase, there was no clear evidence of reversal of any of the findings seen at the end of the dosing phase. This was consistent with the persistence of the hypothyroid state with elevated TSH levels associated with the pharmacological effect of K1-70™.

Administration of K1-70™ to the cynomolgus monkeys resulted in decrease of serum concentrations of T4 and T3 and associated elevation of TSH levels in both males and females (Tables 7 and 8). The microscopic findings in the thyroid showing cystic follicles were consistent with hypothyroidism [19]. Furthermore, vacuolation in the pituitary was noted similar to that observed in the rats. Vacuolation in the pituitary was likely to be related to overstimulation of the thyrotrophs due to hypothyroidism [20]. Decreases in the heart rate in male monkeys was also considered to be due to the hypothyroid state and therefore a pharmacodynamic effect of K1-70™ [23]. Macroscopic and microscopic findings in the thymus (involution/atrophy) were consistent with a hypothyroid state caused by K1-70™ [33]. The changes in the thymus showed recovery at the end of the treatment free phase in the animals treated with K1-70™ by IV infusion and IM treated females. However in IM treated males thymic atrophy/involution was still present at the end of the treatment free phase.

Although the half-life of K1-70™ in the study animals was not determined accurately it ranged from approximately 104 to 263 h in rats and 155 to 425 h in cynomolgus monkeys. This was as expected for monoclonal antibodies [34, 35]. K1-70™ is likely to have a half-life in humans that is similar to that of endogenous IgG of approximately 20 days [36]. Based on these estimates, for the first in human study a follow-up period of up to 100 days, anticipated to cover a period of approximately 5 half-lives, would be appropriate.

In toxicokinetic analyses the NOAEL was not established in the 4-week study in rats. Although no NOAEL was observed in the rat, the toxicological findings were consistent with hypothyroid effects of K1-70™. The NOAEL in the monkeys was 100 mg/kg/dose IV for both males and females. Using the 4-week IV toxicology study data, the human equivalent dose (HED) for the lowest dose evaluated in the rat (15 mg/kg) was 145 mg and using the NOAEL in the primate (100 mg/kg) the HED was 1935 mg. This would indicate a maximum recommended starting dose (MRSD) of 14.5 mg for the first in human study. From the maximum 145 mg HED from the rat toxicology study data the maximum systemic exposure to K1-70™ for the first in human study was determined at C_max_ of 0.031 mg/mL.

Furthermore, a starting dose of K1-70™ for human studies was calculated based upon Food and Drug Administration (FDA) guidance [37] using in vivo effects of IM injections of K1-70™ on thyroid hormone production in rats made hyperthyroid with M22™ [17]. Dose dependent suppression of thyroid activity was observed following IM doses of 50 to 200 µg K1-70™, with no significant effects observed at doses of 4 and 10 µg K1-70™. Taking 10 µg K1-70™ as the minimum effective dose (MED), a HED of 0.645 mg and a starting dose of 0.2 mg were determined. The starting dose of 0.2 mg is approximately one seventieth of the starting dose selected based upon in vivo toxicokinetic data.

The experimentally estimated serum TRAb concentrations in patients with Graves’ disease ranged from 50 to 500 μg/L (TRAb range 1.5–110 U/L) while in patients with autoimmune hypothyroidism with TSHR blocking autoantibodies ranged from 1.7 to 27 mg/L (TRAb range 134–1290 U/L) [38]. The doses of K1-70™ from 0.2 to 100 mg for the first in human study would give serum concentrations of K1-70™ between 0.04 and 20 mg/L which would be well within the concentration range of TRAbs measured in TRAb positive patients.

Overall, the in vivo studies in rats and primates showed that administration of K1-70™ caused hypothyroidism in the study animals which was an expected pharmacodynamic effect. There was no evidence of adverse effects attributed to K1-70™ in the study animals except the decrease in body weight gain, body weight, and food consumption in the rats. All other observations were related to hypothyroidism. This study has not highlighted any systemic or local tolerability concerns following K1-70™ administration via either IV or IM routes. Based on the toxicokinetic analyses in this and previous in vivo studies a starting dose and the maximum systemic exposure of K1-70™ for the first in human study were determined. A Phase 1 clinical trial with K1-70™ in human subjects with Graves’ disease [39] is currently ongoing. This is the first step in development of K1-70™ as a novel drug for improving the management of patients with Graves’ disease, patients with Graves’ ophthalmopathy, patients with thyroid cancer and other patients who would benefit from control of their TSHR activity.

## Abbreviations

ALP: alkaline phosphatase level
AMP: adenosine monophosphate
AUC_0–t_: area under the concentration–time curve over the interval 0 to t, where t is the time of the last measurable concentration
bpm: beats per minute
cGMP: current good manufacturing practice
C_max_: maximum serum concentration
ECG: electrocardiogram
FDA: food and drug administration
HED: human equivalent dose
IgG: immunoglobulin G
IM: intramuscular
IV: intravenous
MED: minimum effective dose
MRSD: maximum recommended starting dose
NOAEL: no observable adverse effect level
SD: standard deviation
t_1/2_: half-life
T3: triiodothyronine
T4: thyroxine
T_max_: time to maximum concentration
TRAb: TSHR autoantibody
TSH: thyroid stimulating hormone
TSHR: TSH receptor
